# Gold nanoparticles synthesized by *Geobacillus* sp. strain ID17 a thermophilic bacterium isolated from Deception Island, Antarctica

**DOI:** 10.1186/1475-2859-12-75

**Published:** 2013-08-06

**Authors:** Daniela N Correa-Llantén, Sebastian A Muñoz-Ibacache, Miguel E Castro, Patricio A Muñoz, Jenny M Blamey

**Affiliations:** 1 Fundación Científica y Cultural Biociencia, José Domingo Cañas 2280, Ñuñoa, Santiago, Chile; 2 Universidad Tecnológica de Chile, Av. Vitacura 10151, Ñuñoa, Santiago, Chile; 3 Doctorado en Biotecnología, Universidad de Santiago de Chile, Avenida Libertador Bernardo O’Higgins 3363, Ñuñoa, Santiago, Chile

**Keywords:** *Geobacillus*, Nanoparticles, Gold, Deception island, Antarctica

## Abstract

**Background:**

The use of microorganisms in the synthesis of nanoparticles emerges as an eco-friendly and exciting approach, for production of nanoparticles due to its low energy requirement, environmental compatibility, reduced costs of manufacture, scalability, and nanoparticle stabilization compared with the chemical synthesis.

**Results:**

The production of gold nanoparticles by the thermophilic bacterium *Geobacillus* sp. strain ID17 is reported in this study. Cells exposed to Au^3+^ turned from colourless into an intense purple colour. This change of colour indicates the accumulation of intracellular gold nanoparticles. Elemental analysis of particles composition was verified using TEM and EDX analysis. The intracellular localization and particles size were verified by TEM showing two different types of particles of predominant quasi-hexagonal shape with size ranging from 5–50 nm. The mayority of them were between 10‒20 nm in size. FT-IR was utilized to characterize the chemical surface of gold nanoparticles. This assay supports the idea of a protein type of compound on the surface of biosynthesized gold nanoparticles. Reductase activity involved in the synthesis of gold nanoparticles has been previously reported to be present in others microorganisms. This reduction using NADH as substrate was tested in ID17. Crude extracts of the microorganism could catalyze the NADH-dependent Au^3+^ reduction.

**Conclusions:**

Our results strongly suggest that the biosynthesis of gold nanoparticles by ID17 is mediated by enzymes and NADH as a cofactor for this biological transformation.

## Introduction

Biological synthesis of nanoparticles appears as a suitable process since it requires less energy, is environmentally safe [1,2], it has low manufacture costs of scalability, and better nanoparticle stabilization, compared to chemically synthesized nanoparticles [3,4].

Nanoparticles have large surface to volume ratio, thus surface related phenomena and properties are drastically affected with slight modification of size, shape and surrounding media [5]. Therefore, the desired optical properties of nanoparticles, depending on the application, can be tuned by generating nanoparticles of defined size and shape in selected media allowing the development of new effective nanomaterials and nanodevices.

Gold nanoparticles show very high chemical reactivity compared to bulk gold, well known for being inert. This kind of nanoparticles has multiple applications in drug-delivery, gene transfer, as bioprobes in cells and for tissue analysis in visualization of micro- and nano-objects, for observation of biological processes at nanoscale [6,7] to enhance electroluminescence and quantum efficiency in organic light emitting diodes [8].

Biosynthesis of gold nanoparticles has been reported in different prokaryotic organisms including *Bacillus subtilis*[9], *Escherichia coli*[10], *Lactobacillus*[11], *Pseudomonas aeruginosa*[12], *Rhodopseudomonas capsulata*[1], but the molecular mechanisms involved in the metal ion reduction taking place for the synthesis of nanoparticles has not yet been established.

Some of these microorganisms can survive and grow even at high metal ion concentrations [4]. They are often exposed to extreme environmental conditions, which forces them to develop specific defense mechanisms to quell such stresses, including the toxicity of foreign metal ions or metalloids [13].

For these reasons and with an applied view we search for microorganisms resistant to high metal concentrations in Deception Island, Antarctica. This place is a complex stratovolcano with a “horseshoe” shape whose central part has a caldera structure. This volcanic island has been very active during the last century: fumarolic emissions, thermal springs and hot soils are evidence of Deception Island’s continuing activity [14], making it an interesting site for searching new thermophilic bacteria.

Here, we report the synthesis of metallic gold nanoparticles by *Geobacillus* sp. strain ID17 mediated by NADH-dependent enzymes that reduce Au^3+^ to elemental gold. Cells exposed to Au^3+^ turned from colourless to an intense purple colour. This change in colour indicates intracellular gold nanoparticles accumulation.

## Results and discussion

The intracellular biosynthesis of gold nanoparticles by a thermophilic bacterium ID17 belonging to genus *Geobacillus* strain isolated from Deception Island [15], was carried out using whole living bacterial cells and cell-free lysates.

**Figure 1:**
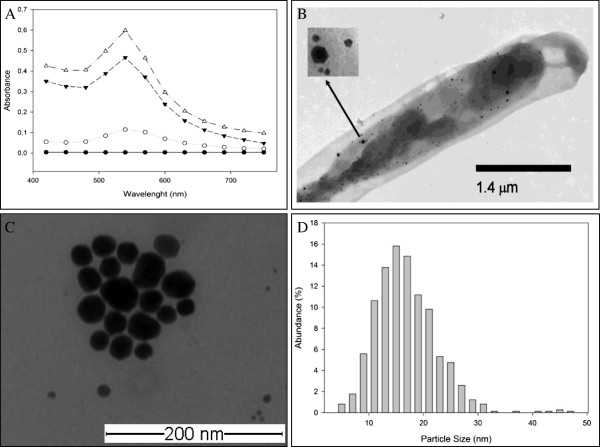
**ID17 and gold nanoparticles production. (A)** Spectra of gold nanoparticles production in the course of time at 0 (●), 3 (○), 9 (▼) and 16 (Δ) h of incubation; **(B)** Transmission electron micrograph. Black spots corresponding to gold nanoparticles and arrow show digital zoom where quasi-hexagonal shapes are shown; **(C)** Gold nanoparticles produced by the microorganism on a copper grille (scale bars correspond to 200 nm). **(D)** Histogram distribution (obtained using Sigma Plot 11.0 software) that shows the particle size distribution (obtained with NIS-Elements D 3.10 software).

**Figure 2:**
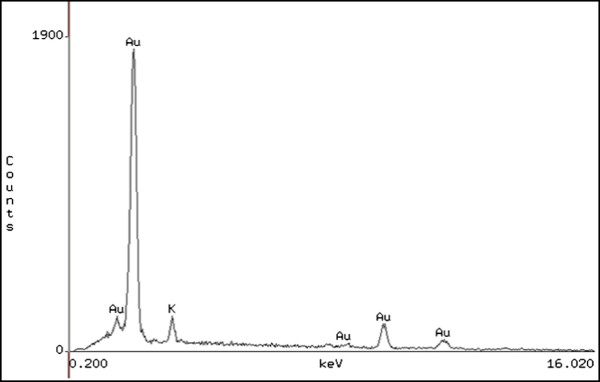
**EDX analysis of gold nanoparticles formed by reduction of HAuCl**_**4 **_**by ID17 cells.** Strong signals from the gold can be observed.

ID17 cells suspended in 20 mM potassium phosphate buffer, pH 7.0 containing 1 mM HAuCl_4_ × 3H_2_O change colour from pale yellow to intense purple colour after incubation for 16 h at 65°C. Colour change of the solution and increments of optical density at 540 nm (Figure 1A) indicate gold nanoparticles formation. This phenomenon has been reported in others biological systems [3,16-19]. In *Stenotrophomonas maltophilia* the production of gold nanoparticles has been correlated with colour change of cell suspension from light yellow to purple [20]. Control experiments showed no change in colour of suspension when cells were not present. Identification, localization and nanoparticles size were verified by transmission electron microscopy (TEM). Microscopic techniques showed the intracellular localization of nanoparticles (Figure 1B) with predominant quasi-hexagonal shape (Figure 1C) and a diameter ranging from 5–50 nm (Figure 1D). Most of them (66%) were between 10–20 nm of size. To study the biosynthesis of nanoparticles, the reaction was monitored at different time intervals over 16 h by UV–vis spectroscopy. EDX spectrum showed that 94.6% of the detected particles by this technique correspond to gold (Figure 2).

**Figure 3:**
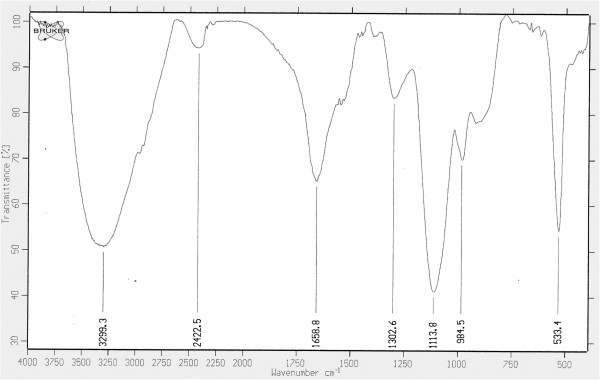
**FTIR spectra of gold nanoparticles synthesized by the reduction of HAuCl**_**4 **_**by ID17.**

Additionally, FT-IR spectroscopy was used to characterize the chemical surface of gold nanoparticles produced by ID17. Figure 3 shows FT-IR of dried powder gold nanoparticles formed after 16 h of reaction. The spectral data recorded revealed two types of vibrations (i.e. stretching and bending) in the wavelength range of 4,000 to 400 cm^-1^. Additionally the data reveal the presence of an amine vibration band 3,299 cm^-1^ representing a primary amine (N-H) stretching, and amide (N-H) bending vibration band at 1,658 cm^-1^. Furthermore, FT-IR spectrum revealed a peak at 2,422 cm^-1^ stretching vibration of aliphatic C-H bonds. C-N stretching vibrations peak were also observed in spectral range of 1,250 to 1,000 cm^-1^. The presence of a peak in 1,302 cm^-1^ suggests the capping agent of biosynthesized nanoparticles posses an aromatic amine group. This FT-IR spectrum supports the idea of protein type of compound on the surface of biosynthesized gold nanoparticles. It is believed that these proteins might be enzymes that reduce the chloroaurate ions and cap the gold nanoparticles formed through the reduction process. It is also possible that different proteins affect capping stabilizing the gold nanoparticles [21].

Reductase activity has been previously reported to be present in other microorganisms involved in nanoparticles synthesis, where the reduction might be initiated by the electron transfer from NADH by a NADH-dependent reductase allowing the reduction of Au^3+^[1]. Similar process has been described for selenium reduction, where the biogenesis of selenium nanoparticles by *Bacillus cereus* involves membrane associated reductases that reduces selenite to elemental selenium through electron shuttle enzymatic metal reduction process [22]. However, the molecular mechanism involved in the reduction of cationic gold is still unclear.

**Figure 4:**
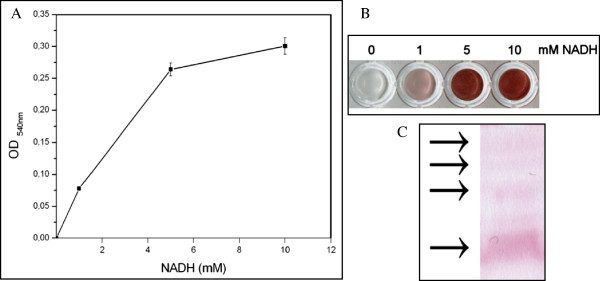
**Evidence of enzymatic Au**^**+3 **^**reduction in ID17. (A)** OD at 540 nm using different concentrations of NADH (1, 5 and 10 mM); **(B)** NADH dependence for the HAuCl_4_ reduction concentration; colour intensity indicates gold nanoparticles formation; **(C)** Zymogram analysis of enzymes (indicated with arrows) involved in Au^+3^ reductase activity.

In order to study the biocatalytic reaction that takes place in the reduction of Au^3+^, crude extracts of ID17 were assayed for their ability to reduce HAuCl_4_ × 3H_2_O. Extracts from ID17 were able to catalyze the NADH-dependent reduction of Au^3+^ (Figure 4A and B). NADH-dependent Au^3+^ reductase activity was dropped approximately in 98% with SDS or proteinase K treatment, reflecting the enzymatic nature of this reaction. The activity was associated to at least four bands present in non denaturing gel PAGE corresponding to proteins in free cells extracts. One of these bands is present in high amount, based on the intensity of the band in a non denaturing gel PAGE (Figure 4C). We are in the process of identifying the enzymes involved in the reduction of Au^+3^ in ID17. It is clear that total reduction of gold observed is not the result of a unique catalytic activity (Figure 4C). Literature report that tellurite reduction in *Aeromonas caviae* ST is exclusively catalyzed by the E3 component (dihydrolipoamide dehydrogenase) of pyruvate dehydrogenase enzyme complex formed by E1, E2 and E3 components [22]. Biosynthesized nanoparticles display good surface bioactivity for their association to proteins, a remarkable property for biomedicine and health care applications.

Regarding nanoparticles form, the quasi-hexagonal crystal shape is the predominant form under the experimental conditions reported in this work. Studies reveal that the morphology and dimensions of nanoparticles are strongly dependent of biosynthesis conditions, such as temperature, concentration of metallic salt, pH [23]. He et al. [24] demonstrated that the bacterium *Rhodopseudomonas capsulate* produces gold nanoparticles of different sizes and shapes when was incubated with HAuCl_4_ salts, was exposed to distinct pH values (spherical at pH 7.0 and nanoplates at pH 4.0), converting it in the most important factor to control these parameters. We think that the influence of different reductases associated to the biosynthesis could result in differences of shape but our experimental results cannot be conclusive to probe this hypothesis.

Gold nanoparticles shape and size are very important factors for the immunological response *in vitro* and *in vivo*[24]. It has been reported that 40 nm spherical nanoparticles induce the highest level of specific antibodies against West Nile virus, while rod nanoparticles lead only to 50% of the antibody production against the virus, indicating the high variability of immunological responses when different nanoparticle shapes were used [24].

Moreover, gold nanoparticles have different biological applications that include immunostaining of specific molecules or compartments of cells by antibodies. Using the previous concept, gold nanoparticles can be useful as contrast agents for X-rays. They also can be used as vehicle for delivery of molecules into cells, where the molecules are absorbed on the surface of gold nanoparticles and the whole conjugate is introduced into the cells. Finally the intracellular accumulation of gold nanoparticles by *Geobacillus* sp. provides also a potential application of this microorganism in bioremediation of gold-bearing waste. Even more, gold nanoparticles can be used as a heat source, if they absorb light by their inner electrons and dissipating heat when they relax [25].

## Conclusion

ID17, a thermophilic bacterium belonging to the genus *Geobacillus*, has the ability to biosynthesize gold nanoparticles, which are intracellularly accumulated. This property is present in whole cells and in free cell extracts indicating that this process is probably enzymatically mediated, due to the requirement of NADH as cofactor for this biological transformation.

## Methods

### Bacterial strains and culture conditions

ID17 was isolated as described by Muñoz *et al*. (2011) from environmental samples from Deception Island (Antarctica). Cells were grown in BS medium (0.3 g/L NaCl; yeast extract 0.15 g/L; 0.3 g/L tryptone) for 16 h at 65°C and pH 7.0. The sequence of the 16S rDNA of the isolate was deposited in Genbank nucleotide sequences databank under accession number U366067 [15].

### Biosynthesis of gold nanoparticles

Assays were carried out using whole living bacterial cells and cell-free lysates. For the biosynthesis of gold nanoparticles, cells from culture with OD_600_ ~ 0,5 were harvested by centrifugation at 6,000 rpm for 5 min and washed three times with sterile water.

### Using whole living bacterial cells assay

The harvested bacteria were suspended in 10 mL solution of 20 mM potassium phosphate buffer, pH 7.0 containing 1 mM hydrogen tetrachloroaurate (HAuCl_4_ × 3H_2_O, Sigma). The solution was incubated at 65°C for 16 h. Bacterial-gold nanoparticles were recovered as described by Fesharaki *et al.*, [26]. Cell debris was discarded and nanoparticles were characterized.

### Using cell-free lysates assay

Cell pellet was suspended in 20 mM potassium phosphate buffer, pH 7.0 and disrupted by sonication for 10 min at level 3 in a Sonifier 450 (Branson). Cell debris was discarded and the supernatant was kept at 4°C. Protein concentration was determined as described by Bradford using bovine serum albumin as standard [27].

Crude extracts were assayed for their ability to reduce hydrogen tetrachloroaurate. To determine the presence of the intracellular enzymes involved in the biological transformation, 100 μg of protein were added in 20 mM potassium phosphate buffer, pH 7.0 containing 1 mM hydrogen tetrachloroaurate and NADH in a range of 0–10 mM. Final volume of reaction was 1 mL. Reactions were always started by the addition of free cell extract and incubated at 65°C for 30 min. The reaction was followed by measuring the change in absorbance at 540 nm in a spectrophotometer.

### Zymogram

To identify the purple deposits as reduced Au^3+^, free cell extract was subjected to non-denaturing polyacrylamide (10%) gel electrophoresis (PAGE) to detect Au^3+^ reduction activity. The native gel containing the proteins present in the crude extract was immersed in 20 mM potassium phosphate buffer, pH 7.0 containing 1 mM HAuCl_4_ × 3H_2_O, 5 mM NADH and incubated at 65°C for 1 h. Reduction activity was detected by the change of colour from colourless to purple.

### Gold nanoparticles characterization

#### Spectra

Gold nanoparticles spectra were performed scanning from 400 to 900 nm using UV/visible spectrophotometer (Shimadzu UV-1700) and 1.0 cm light-path length cuvette.

#### Transmission electron microscopy measurements (TEM)

10 μL of sample were dropped on a carbon coated copper grid and dried at room temperature. For the size determination of nanoparticles, bacteria were recovered as described by Fesharaki *et al.*, [26]. TEM measurements were performed on a Philips Tecnai 12 Bio Twin TEM operating at 200 kV. Sizes were obtained with NIS-Elements D 3.10 software. Histogram distribution of sizes was constructed with Sigma Plot 11.0 software.

#### Energy-Dispersive X-Ray Microanalysis (EDX)

Elemental analyses of nanoparticles were conducted by using energy*-*dispersive X*-*ray microanalysis. This was carried out using a scanning electron microscope (SEM) Jeol 5410 equipped with an energy dispersive X-ray spectrometer.

#### Fourier-Transform Infrared (FT-IR) Chemical Analysis

For Fourier-Transform Infra-Red spectroscopy measurements, the nanoparticles extracted from bacteria were freeze dried and diluted with potassium bromide in the ratio of 1:100. The FT-IR spectra of samples were recorded on a FT-IR instrument (IFS66V, Bruker). All measurements were carried out in the range of 400–4,000 cm^-1^ at a resolution of 4.0 cm^-1^.

## Competing interests

The authors declare that they have no competing interests.

## Authors’ contributions

DC, MC, and JB carried out the design of the study, PM purified the microorganism and DC, SM and MC performed the majority of the experimental work. All authors participated in writing and critical review of the manuscript. All authors have read and approved the manuscript.
